# Anxiety in youth with and without specific learning disorders: exploring the relationships with inhibitory control, perfectionism, and self-conscious emotions

**DOI:** 10.3389/fnbeh.2025.1536192

**Published:** 2025-03-10

**Authors:** Rachele Lievore, Ramona Cardillo, Irene Cristina Mammarella

**Affiliations:** ^1^Department of Developmental Psychology and Socialization, School of Psychology, University of Padua, Padua, Italy; ^2^Department of Women’s and Children’s Health, University of Padua, Padua, Italy

**Keywords:** anxiety, specific learning disorders, inhibition, perfectionism, shame, guilt

## Abstract

**Introduction:**

Since early research on Specific Learning Disorders (SLD), their relationship with emotional issues have been recognized, although emotional factors have received less attention compared to the cognitive processes related with academic achievement.

**Methods:**

This study aimed to investigate mechanisms that may increase vulnerability to generalized and social anxiety in youth with SLD, compared to their non-diagnosed peers. We examined cognitive factors (inhibitory control), personality traits (self-oriented, socially prescribed perfectionism), and self-conscious emotions (shame, guilt). The sample included 134 individuals aged 10 to 16, divided into two groups: 67 with SLD and 67 without diagnoses, matched by age, sex, and IQ. Participants completed questionnaires on anxiety, perfectionism, and selfconscious emotions, alongside an inhibitory control task.

**Results:**

Findings revealed that those with SLD reported higher generalized and social anxiety, poorer inhibitory control, greater socially prescribed perfectionism, and more shame than nondiagnosed peers. Socially prescribed perfectionism was found to increase the risk of generalized anxiety in participants with SLD, while both socially prescribed perfectionism and shame were predictors of social anxiety across both groups. Finally, self-oriented perfectionism seemed to be associated with lower social anxiety in the SLD group.

**Discussion:**

These findings suggest that interventions should address risk and protective factors, focusing on reducing anxiety and fostering adaptive self-regulation strategies.

## Introduction

Specific Learning Disorders (SLD) are defined by difficulties in learning and academic skills, such as reading, writing, and mathematics, despite average (or above) intellectual abilities. These difficulties significantly interfere with school performance and/or daily functioning (DSM-5; American Psychiatric Association [APA], 2013). Both as a consequence of, and a reinforcing cyclical trigger of learning challenges, students with SLD often struggle with lower self-esteem (Alexander-Passe, 2006; Novita, 2016; Zuppardo et al., 2023) and self-efficacy (Elgendi et al., 2021), excessive fear of being negatively evaluated by others (Filippello et al., 2020; Lufi et al., 2004; Mammarella et al., 2016), along with social functioning problems (Lievore et al., 2024a; Parhiala et al., 2015). All these aspects may have an impact on the emotional functioning of young people with SLD, potentially leading to increased vulnerability to internalizing symptoms and heightened anxiety levels (Carroll and Iles, 2006; Livingston et al., 2018; Nelson and Harwood, 2011).

A meta-analysis published on 2011 revealed that approximately 70% of youth with SLD experience higher levels of anxiety symptoms compared to their non-diagnosed peers (Nelson and Harwood, 2011), although no strong genetic component has been observed linking the two conditions (Whitehouse et al., 2009). Many studies found evidence for anxiety symptoms in children (Haft et al., 2019; Mammarella et al., 2016; Novita, 2016; Wilmot et al., 2024; Zuppardo et al., 2023) and adolescents with SLD (Giovagnoli et al., 2020; Goldston et al., 2007; Scorza et al., 2018; Wilson et al., 2009), which may even persist into adulthood (Goldberg et al., 2003; Lufi et al., 2004; Potard et al., 2022; Wilson et al., 2009). Specifically, research distinguishing between different types of anxiety has demonstrated a higher occurrence of generalized and social anxiety in SLD (Carroll et al., 2005; Carroll and Iles, 2006; Goldston et al., 2007; Mammarella et al., 2016; Thaler et al., 2010). Indeed, the difficulties experienced at school, combined with maladaptive coping strategies, may generalize to other areas of daily life, leading to the development of generalized anxiety symptoms, such as excessive worry, trembling, restlessness, and tension (Kajastus et al., 2024). Furthermore, the likelihood of developing social anxiety symptoms may be prompted by negative feedback on academic performance from teachers and may be exacerbated by repetitive failures experienced at school in front of peers (Lievore et al., 2024b; Novita, 2016; Sahoo et al., 2015; Wilmot et al., 2023). In this sense, the excessive worry about being negatively judged and the comparison with other students may contribute to the development of social worries.

A well-known cognitive vulnerability factor for the development of anxiety is inhibitory control, an executive function involving the ability of suppressing a dominant response and controlling interference (Schachar and Logan, 1990). A lack of inhibitory ability and poor executive attentional control is interactively associated with heightened worry responses (Ansari and Derakshan, 2011; Myles et al., 2020), consistent with generalized anxiety (Hallion et al., 2017; Hirsch and Mathews, 2012). However, some studies found that children with high levels of executive inhibition were significantly more at risk for developing social anxiety, especially those characterized by social reticence (Thorell et al., 2004; Troller-Renfree et al., 2019; White et al., 2011). The relationship between inhibitory control and anxiety may be particularly relevant in people with SLD, as challenges in executive functions can make it more difficult to manage anxiety (and vice versa), especially in academic and social contexts (Alesi et al., 2024; Margolis and Liu, 2023; Wang et al., 2024). In fact, most of the studies showed a worse ability to hinder an impulsive response in SLD compared to non-diagnosed peers (Agostini et al., 2022; Capodieci et al., 2023; Crisci et al., 2021; Mirabella, 2021). However, no studies investigated the relationship between inhibitory control and the occurrence of anxiety in SLD.

In addition to vulnerabilities in inhibitory control, it may be crucial to investigate personality traits, such as the tendency toward perfectionism, and emotional characteristics, such as a predisposition to experiencing shame and guilt. This can help to understand how the influence of expectations and moral standards may heighten anxiety in young people with SLD.

The relationship between perfectionism and anxiety is complex and multifaceted (Burgess and DiBartolo, 2016). Perfectionism has been conceptualized as having two main dimensions (Flett et al., 2016): self-oriented perfectionism, which refers to setting exceptionally high personal standards and being driven to achieve them, and socially prescribed perfectionism, which involves the belief that others expect perfection from oneself. Since perfectionism implies a combination of excessively high standards and an overly critical evaluative style, it appears to play an important role in the maintenance of multiple psychopathological states (Flett and Hewitt, 2014; Hamachek, 1978; Shafran et al., 2002). In regard to anxiety, perfectionism often involves setting unattainable goals and an intense fear of failure, leading to chronic worry, a hallmark of generalized anxiety (Burgess and DiBartolo, 2016; Essau et al., 2008). With respect to social anxiety, perfectionists may experience significant performance anxiety in social situations, driven by the fear that their interactions will fall short of their high standards, especially when these standards are shaped by societal expectations (Hewitt et al., 2002; Laurenti et al., 2008). In fact, social anxiety has been shown to be more related to the socially prescribed dimension of perfectionism (Laurenti et al., 2008; Wheeler et al., 2011).

The investigation of perfectionism traits can be useful in the case of SLD, to understand whether the difficulties they experience may lead to significant distress due to their inability to meet self- or externally imposed (by parents or school) goals. Indeed, perfectionism can lead to increased generalized anxiety in SLD, stemming from the frustration of failing to meet both personal and parental expectations (Scott, 2003). Despite the clinical and educational importance of the topic, few studies have explored perfectionism in individuals with SLD and its potential relationship with anxiety symptoms. A recent study (Stoeber and Rountree, 2021) revealed that socially prescribed perfectionism is a dysfunctional form that predicts greater psychological maladjustment in SLD, while self-oriented perfectionism shows a more mixed profile (Stoeber et al., 2009). Although self-oriented perfectionism is linked to increased self-stigma and maladaptive coping, it can also positively influence adaptive coping directly (Stoeber and Rountree, 2021). In this sense, self-oriented perfectionism may be positively related to academic performance and helpful academic outcomes (Osenk et al., 2020). Conversely, when the high standards are not met, perfectionists often experience feelings of inadequacy, which may contribute to a pervasive sense of shame and guilt (Stoeber et al., 2007; Tangney, 2002).

Shame and guilt are defined as self-conscious emotions because they are emotions that fundamentally involve a (negative) evaluation of the self (Tangney, 2002; Tangney and Dearing, 2003; Tracy et al., 2013). While shame encompasses a deeply painful assessment of the self, leading to feelings of worthlessness and incompetence, guilt is a more focused emotion that arises from a negative evaluation of a specific behavior. Guilt typically brings feelings of regret; thus, it can also serve as a catalyst for individuals to make amends. In contrast, shame drives behaviors of defensiveness and avoidance, potentially acting as a natural mechanism for expressing submission. Dysregulations of self-conscious emotions have been associated with various types of psychopathological outcomes (Muris and Meesters, 2014), among which anxiety (Hendriks et al., 2022; Muris et al., 2015). In particular, generalized and social anxiety have been found to be more closely related to the tendency to experience shame, rather than guilt (Austin and Richards, 2001; Fergus et al., 2010; Pineles et al., 2006; Swee et al., 2021). However, some studies have revealed a significant relationship between anxiety symptoms and guilt (Gilbert, 2000; Hendriks et al., 2022; Muris et al., 2016). Theoretically, it is logical to connect these self-conscious emotions to anxiety due to shared characteristics, including negative self-evaluation, the fear of losing social status, feelings of inferiority, low self-esteem, and avoidance behavior. Moreover, research has indicated that students experience shame in a variety of academic settings, and this might negatively affect their self-regulation at school, their motivation to learn and their achievements (Pekrun, 2006; Sullins et al., 2024). Research on students with SLD is limited; however, they seem to have more negative self-perceptions and lower self-esteem, which could lead to greater levels of shame and guilt (Alexander-Passe, 2006; Gibby-Leversuch et al., 2021).

### The present study

The main aim of the current study was to explore the vulnerability mechanisms associated with the onset of generalized and social anxiety in youth with SLD compared to non-diagnosed peers, focusing on cognitive factors (i.e., inhibitory control), personality dispositions (i.e., self-oriented and socially prescribed perfectionism), and self-conscious emotions (i.e., shame and guilt).

The first aim was to compare young individuals with and without SLD, matched for age, sex and full-scale intelligence quotient (FIQ), in terms of reported levels of generalized and social anxiety. It was hypothesized that participants with SLD will report higher levels of generalized and social anxiety compared to those without SLD (Carroll et al., 2005; Carroll and Iles, 2006; Goldston et al., 2007; Mammarella et al., 2016; Thaler et al., 2010).

Second, the current study aimed to compare vulnerability factors that may contribute to increased levels of anxiety symptoms in students with and without SLD. We considered measures of inhibitory control, self-oriented and socially prescribed perfectionism, and the tendency to experience shame and guilt. Participants with SLD were expected to show a worse inhibitory control than those without SLD (Agostini et al., 2022; Capodieci et al., 2023; Crisci et al., 2021; Mirabella, 2021). Moreover, taking a more exploratory approach due to the limited literature, we expected that students with SLD will have higher levels of socially prescribed perfectionism, for the significant role of other people’s expectations on their emotional state and coping strategies (Stoeber and Rountree, 2021). Lastly, higher levels of shame and guilt were hypothesized in participants with SLD compared to their non-diagnosed peers, due to the possible negative academic and social experiences (Alexander-Passe, 2006; Gibby-Leversuch et al., 2021).

Our third aim was to examine whether and how these vulnerability mechanisms might be associated with the levels of generalized and social anxiety in participants with SLD compared to non-diagnosed peers, controlling for age and sex. We expected that poor inhibitory control may be associated with greater levels of generalized anxiety (Hallion et al., 2017; Hirsch and Mathews, 2012), especially in the SLD group due to well-known difficulties in inhibition and emotional regulation. However, based on previous findings (Thorell et al., 2004; Troller-Renfree et al., 2019; White et al., 2011), we could also suppose that higher levels of social anxiety may be associated with higher inhibitory control. Furthermore, it was reasonable to assume that greater socially prescribed perfectionism (Laurenti et al., 2008; Wheeler et al., 2011) and proneness to shame (Fergus et al., 2010; Swee et al., 2021) could be linked to higher levels of social anxiety in the SLD group, owing to the excessive worry of negative evaluation commonly observed in this condition (Filippello et al., 2020; Lufi et al., 2004; Mammarella et al., 2016; Sullins et al., 2024). In contrast, we expected that higher levels of self-oriented perfectionism could be associated with better coping outcomes, consistent with lower levels of anxiety symptoms in students with SLD (Stoeber and Rountree, 2021).

## Methods

### Participants

The study involved 134 participants aged between 10 and 16 years old divided into two groups: 67 (38 boys) participants with Specific Learning Disorders (SLD) and 67 (30 boys) without any diagnosis (ND). The two groups did not differ statistically in chronological age, *F*(1, 132) = −1.22, *p* = 0.27, *Cohen’s d* = −0.19, sex distribution, *Χ^2^* = 1.91, *df* = 1, *p* = 0.17, or full-scale IQ, *F*(1, 132) = 2.67, *p* = 0.11, *Cohen’s d* = −0.28.

All participants in the clinical group had been previously diagnosed with SLD, according to the DSM-IV-TR, the DSM-5 (American Psychiatric Association, 2000; American Psychiatric Association, 2013) or ICD10 (World Health Organization, 1992) criteria, with major impairments in both reading and math abilities. Diagnoses of SLD were also confirmed by implementing some age-appropriate subtests assessing reading and math competencies, such as reading lists of words and pseudo-words (MT-Avanzate-3, Cornoldi et al., 2017; DDE-2, Sartori et al., 2007) and mental calculation (AC-MT-3, Cornoldi et al., 2020; MT-Avanzate-3, Cornoldi et al., 2017). Approximately 58% (*n* = 39) of participants with SLD exhibited a combined profile with difficulties in two or more areas of learning, 19% (*n* = 13) in reading, 12% (*n* = 8) in writing, and 10% (*n* = 7) in mathematics. The control group consisted of healthy individuals without any diagnoses of psychiatric, neurological, or neurodevelopmental disorders.

Participants from both groups were included in this study only if they achieved a full-scale IQ standard score of at least 85 on the Wechsler Intelligence Scales (WISC IV; Wechsler, 2003). The two groups differed statistically in both reading (errors and speed – measured in seconds – in words and pseudowords) and math (mental calculation accuracy and response times) subtests, with the SLD group showing greater impairment than the ND group. A summary of the participants’ characteristics is shown in Table 1.

**Table 1:** Descriptive statistics and statistical analyses on the screening variables.

Measures	SLD (*n* = 67)		ND (*n* = 67)		*F*(1, 132)	*p*	*Cohen’s d*
Sex M:F	38:29		30:37				
	M	SD	M	SD			
Age	12.42	1.47	12.72	1.65	1.22	0.27	−0.19
IQ	105.54	10.04	108.61	11.66	2.67	0.11	−0.28
Reading (z-score)							
Words (errors)	2.18	1.20	0.18	1.05	46.72	**<0.001**	1.76
Words (seconds)	2.11	1.21	0.27	0.97	49.89	**<0.001**	1.68
Pseudo-words (errors)	1.28	1.39	−0.03	1.16	33.57	**<0.001**	1.02
Pseudo-words (seconds)	1.92	1.22	0.50	1.16	33.52	**<0.001**	1.19
Math (z-score)							
Mental calculation (accuracy)	−1.19	1.07	0.39	1.04	75.41	**<0.001**	−1.50
Mental calculation (response times)	1.57	1.29	0.20	1.02	44.76	**<0.001**	1.17

All reading, and math subtests are expressed in standardized z scores. M, mean; SD, standard deviation; SLD, Specific Learning Disorders; ND, Non-Diagnosed; IQ, full-scale intelligence quotient. Statistically significant results are reported in bold.

Participants currently taking psychotropic medications, having other known chronic medical or genetic conditions, a history of neurological diseases, comorbid psychopathologies, or certified physical and intellectual disabilities were excluded. All participants were native Italian speakers, and none had any visual or hearing impairments.

## Materials

### Anxiety

The children’s self-report version of the *Multidimensional Anxiety Scale for Children* (MASC-2; March, 2012; Italian version, Paloscia et al., 2017) was administered. Specifically, the dimensions related to the Generalized Anxiety Disorder (GAD) index (10 items) and the social anxiety (9 items), which include humiliation/rejection and performance fears, have been considered. Participants are required to rate the frequency of specific thoughts, behaviors or feelings. Responses are rated on a scale from 0 (“never”) to 3 (“often”). Raw scores are then transformed into T scores using age- and sex-specific normative data, where a T score of 60 determines a clinical cut-off for anxiety.

### Inhibitory control

Inhibitory control was assessed using a computerized go/no-go task (Lievore et al., 2024a). The task consisted of 120 trials divided into two blocks of 60 trials each, with a break in between. During each trial, one of four colored dots (blue, red, yellow, or green) appeared on a computer screen. In the first block, participants were instructed to press the spacebar as quickly as possible when a blue dot appeared (target; go trials) and to refrain from responding when a dot of any other color was displayed (non-target; no-go trials). In the second block, the task was reversed: participants were instructed to press the spacebar when a dot of any color except blue appeared (target; go trials) and to withhold their response when a blue dot appeared (non-target; no-go trials). Performance on the go trials measured attention, while errors on the no-go trials assessed inhibitory control. Within each block, stimuli were presented in a random order, with targets appearing in 25% of the trials. Each trial began after a 2000 ms intertrial interval. Before the main task, participants completed eight practice trials, during which they received feedback on their performance (“correct,” “incorrect,” or “too slow” if they failed to respond within 2000 ms). Errors on the no-go trials (i.e., responses to non-target stimuli) served as the primary measure of inhibitory control. The higher the score, the poorer the inhibitory control.

### Perfectionism

The *Child–Adolescent Perfectionism Scale* (CAPS; Flett et al., 2016) is a 22-item questionnaire in which participants are required to rate how much they agree with the given statements. The possible answers for each sentence are rated on a 5-point Likert scale (1 = “False - not at all true of me,” 2 = “Mostly false,” 3 = “Neither true nor false,” 4 = “Mostly true,” 5 = “Very true of me”). The CAPS has two dimensions: (a) self-oriented perfectionism (i.e., having high personal standards and being strongly motivated to achieve them) and (b) socially prescribed perfectionism (i.e., having the belief or perception that others expect perfection from oneself). Examples of item are: “I try to be perfect in everything I do” (self-oriented perfectionism) and “My teachers expect my work to be perfect” (socially prescribed perfectionism). Higher scores indicate higher tendency to perfectionism.

### Shame and guilt

The *Test of Self-Conscious Affect* (TOSCA; Tangney and Dearing, 2003; Tangney et al., 1990, 1991) was employed to assess susceptibility to self-conscious emotions, making it suitable for children aged eight through adolescence. The test consisted of 15 scenarios based on real-life situations, which are read aloud to the participants, accompanied by illustrations depicting the events. Following each scenario, four or five statements are presented that explore the tendency toward specific self-conscious emotions (such as guilt, shame, hubristic pride, or authentic pride) or mental states (like externalization or detachment). Participants are asked to imagine themselves in the situation and rate how likely they would be to experience each emotion or mental state on a five-point scale (1 = “Not Likely,” 2 = “Unlikely,” 3 = “Maybe,” 4 = “Likely,” 5 = “Very Likely”). An example of scenario is “You get a test back in school and you did not go well” and examples of response statements are “I’d feel stupid” (shame) and “I’d feel that I should have done better. I should have studied more” (guilt). For this study, the tendencies to experience shame and guilt were considered, where higher ratings indicated greater proneness to these self-conscious emotions.

## Procedure

The study was approved by the ethics review board of the Authors’ institution and adheres to the APA ethical standards. The sample was recruited from clinical centers (SLD) and schools (ND). Following discussions with clinical centers’ directors about the research project, families of participants with SLD were contacted to assess their interest and willingness to participate. Upon receiving consent, permission was requested to provide the experimenter with their contact details. After obtaining the written consent of the participants’ parents to their participation in the study, the SLD group underwent evaluation at child and adolescent psychiatric service centers where they were referred. Instead, the ND participants were engaged and examined individually at their respective schools during regular school hours, outside the classroom, so as not to disturb the continuation of the lesson.

The study involved two sessions lasting approximately 45 min each. In the screening phase, the IQ was calculated and only participants who scored above 85 were included; reading and math competences were also evaluated in this initial phase. The experimental phase included the questionnaires on anxiety (generalized, social), personality traits (self-oriented, socially prescribed perfectionism), and self-conscious emotions (shame, guilt), and a computerized test for inhibitory control. The order of administration was counterbalanced for each participant. The computerized task was created and administered using PsychoPy3 (Peirce et al., 2019) and a laptop computer with a 15-inch LCD screen.

### Statistical approach

A series of univariate ANOVAs were performed to estimate differences between the two groups (SLD, ND) in the measures of interest, with age as a covariate. Effect sizes were computed using the *partial η^2^*, which expresses the magnitude of the difference between two groups’ means. Spearman’s correlations divided by group (SLD, ND) are reported in the supplementary materials (Supplementary Table S1).

Two hierarchical linear regression models were run to investigate the association between the dependent variables (GAD index and social anxiety) and the predictors (inhibitory control, self-oriented perfectionism, socially prescribed perfectionism, shame, guilt). The interaction effects with the group’s membership were also considered. In the first step, age and sex were included as covariates, to control for their effect. In the second step, the group was added; in the third, inhibitory control; in the fourth, self-oriented perfectionism, socially prescribed perfectionism, shame and guilt. The interactive effect of group (SLD, ND) with the variables was included in the fifth step. The residual errors of both regression models were normally distributed, as confirmed by the Shapiro–Wilk test and visual inspection of the Q-Q plot.

The best model was then selected using information-theoretic (I-T) approaches (Burnham et al., 2011), considering the Akaike information criterion (*AIC*), the *R^2^* and the adjusted *R^2^* (*Adj R^2^*). The *AIC* is an estimator of prediction error and therefore of the relative quality of statistical models for a given set of data: a lower *AIC* indicates a better model. *R^2^* assesses the proportion of variance in the dependent variable explained by the model, with higher values indicating better explanatory power. The *Adj R^2^* is a modified version of *R^2^* that adjusts for the number of predictors in a regression model and allows for comparing models with a different number of predictors (Miles, 2005). Moreover, the log-likelihood (*logLik*) and the *RMSE* (Root Mean Square Error) were also calculated: the *logLik* reflects the probability of the data given the model, where higher values suggest a better fit; the *RMSE* measures the average deviation of predictions from observed values, indicating model accuracy.

Data were analyzed using R version 4.3.2 (R Core Team, 2023). The following R packages were used: “sjPlot” package (Lüdecke, 2013) for the correlation matrix, “effectsize” for computing *partial η^2^* (Ben-Shachar et al., 2020), “lme4” package (Bates et al., 2015) to run the regression models, and “effects” package (Fox and Weisberg, 2018) for graphical effects.

## Results

### Comparison between groups

The two groups statistically differed in reported levels of anxiety, with participants with SLD referring higher levels of GAD index, *F*(1, 132) = 9.24, *p* = 0.003, *partial η^2^* = 0.07, and social anxiety, *F*(1, 132) = 8.68, *p* = 0.004, *partial η^2^* = 0.06, as compared to ND peers. Moreover, the SLD group performed statistically worse in the inhibitory control task than the ND participants, *F*(1, 132) = 13.94, *p* < 0.001, *partial η^2^* = 0.10. As concerns perfectionism, the two groups were statistically different in the socially prescribed perfectionism, *F*(1, 132) = 5.95, *p* = 0.01, *partial η^2^* = 0.04, with participants with SLD reporting higher levels than ND peers; however, no significant difference emerged between the groups in the self-oriented perfectionism, *F*(1, 132) = 2.71, *p* = 0.10, *partial η^2^* = 0.02. Participants with SLD also reported greater proneness to experience shame than ND peers, *F*(1, 132) = 7.78, *p* = 0.006, *partial η^2^* = 0.06. No difference between groups was found for guilt-proneness, *F*(1, 132) = 0.19, *p* = 0.66, *partial η^2^* = 0.003. Table 2 displays descriptive statistics and statistical comparisons between the groups across all considered measures.

**Table 2:** Descriptive statistics and statistical comparisons between groups on the measures of interest, with age as a covariate.

Measures	SLD		ND		*F*(1, 132)	*p*	Partial η^2^
	M	SD	M	SD			
GAD index	54.57	9.09	50.33	8.04	9.24	**0.003**	0.07
Social anxiety	54.57	9.67	50.06	8.77	8.68	**0.004**	0.06
Inhibitory control	4.16	2.69	2.43	2.32	13.94	**<0.001**	0.10
Self-oriented perfectionism	36.09	8.93	34.04	8.15	2.71	0.10	0.02
Socially prescribed perfectionism	26.75	8.47	23.33	8.38	5.95	**0.01**	0.04
Shame	43.42	9.42	39.07	8.80	7.78	**0.006**	0.06
Guilt	57.33	10.19	56.88	9.75	0.19	0.66	0.003

M, mean; SD, standard deviation; SLD, Specific Learning Disorders; ND, Non-Diagnosed; GAD index, Generalized Anxiety Disorder index. Statistically significant results are reported in bold.

### Regression models

To investigate the possible contribution of the investigated variables on the GAD index and social anxiety, hierarchical regression models were run by sequentially entering predictors (and their interaction with group) in different steps, considering age and sex as covariates. Table 3 includes the two hierarchical regression models with GAD index and social anxiety as dependent variables. Models’ comparison is shown in Table 4 (*AIC, Δ°AIC, logLik, RMSE, R^2^, adj R^2^*).

**Table 3:** Hierarchical regression models with generalized anxiety disorder (GAD) and social anxiety indexes as dependent variables.

Models	GAD index					Social anxiety				
	Std estimate	*β*	*SE*	*t*	*p*	Std estimate	*β*	*SE*	*t*	*p*
Model 1										
Age	0.10	0.05	0.04	1.15	0.25	0.07	0.03	0.04	0.78	0.44
Sex	0.05	0.95	1.53	0.62	0.54	0.02	0.42	1.65	0.25	0.80
Model 2										
Age	0.13	0.06	0.04	1.55	0.12	0.10	0.05	0.04	1.17	0.24
Sex	0.02	0.38	1.49	0.25	0.80	−0.01	−0.19	1.62	−0.12	0.90
Group	−0.25	−4.47	1.50	−2.97	**0.003**	−0.25	−4.75	1.62	−2.93	**0.004**
Model 3										
Age	0.10	0.04	0.04	1.12	0.26	0.07	0.04	0.04	0.87	0.39
Sex	0.04	0.66	1.49	0.44	0.65	0.001	0.02	1.63	0.01	0.99
Group	−0.29	−5.22	1.56	−3.34	**0.001**	−0.28	−5.31	1.70	−3.12	**0.002**
Inhibitory control	−0.15	−0.49	0.30	−1.63	0.10	−0.10	−0.36	0.33	−1.10	0.27
Model 4										
Age	0.07	0.03	0.04	0.80	0.42	0.07	0.03	0.04	0.88	0.38
Sex	0.05	0.92	1.45	0.63	0.53	−0.006	−0.12	1.48	−0.08	0.94
Group	−0.21	−3.76	1.58	−2.39	**0.02**	−0.15	−2.94	1.60	−1.83	0.07
Inhibitory control	−0.14	−0.48	0.29	−1.61	0.11	−0.07	−0.26	0.30	−0.88	0.38
Self-oriented perfectionism	−0.09	−0.09	0.09	−0.99	0.32	−0.16	−0.18	0.09	−1.99	**0.04**
Socially prescribed perfectionism	0.19	0.20	0.09	2.28	**0.02**	0.24	0.27	0.09	2.97	**0.003**
Shame	0.18	0.17	0.09	1.81	0.07	0.37	0.37	0.10	3.85	**<0.001**
Guilt	0.16	0.14	0.09	1.51	0.13	0.05	0.04	0.09	0.48	0.63
Model 5										
Age	0.05	0.02	0.04	0.61	0.54	0.07	0.04	0.04	0.93	0.35
Sex	0.05	0.87	1.46	0.59	0.55	−0.003	−0.06	1.51	−0.04	0.97
Group	0.10	1.81	10.76	0.17	0.87	0.03	0.51	11.11	0.05	0.96
Inhibitory control	−0.20	−0.67	0.38	−1.79	−0.08	−0.06	−0.22	0.39	−0.56	0.57
Self-oriented perfectionism	−0.18	−0.18	0.11	−1.59	0.11	−0.29	−0.32	0.12	−2.74	**0.007**
Socially prescribed perfectionism	0.40	0.41	0.12	3.44	**<0.001**	0.30	0.33	0.12	2.70	**0.008**
Shame	0.26	0.25	0.15	1.65	0.10	0.45	0.46	0.15	2.94	**0.004**
Guilt	0.11	0.09	0.14	0.68	0.49	0.03	0.03	0.14	0.22	0.82
Inhibitory control*Group	0.05	0.22	0.58	0.38	0.70	−0.06	−0.30	0.59	−0.51	0.61
Self-oriented perfectionism*Group	0.61	0.29	0.18	1.64	0.10	0.77	0.41	0.19	2.17	**0.03**
Socially prescribed perfectionism*Group	−0.72	−0.49	0.18	−2.67	**0.008**	−0.29	−0.21	0.19	−1.14	0.26
Shame*Group	−0.14	−0.06	0.19	−0.31	0.76	−0.32	−0.15	0.21	−0.71	0.48
Guilt*Group	−0.11	−0.03	0.19	−0.18	0.85	−0.29	−0.09	0.19	−0.49	0.62

GAD index, Generalized Anxiety Disorder index. Statistically significant results are reported in bold.

**Table 4:** Models’ fit indexes with GAD and social anxiety indexes as dependent variables.

Models	GAD index						Social anxiety					
	*AIC*	*Δ*°*AIC*	*logLik*	*RMSE*	*R^2^*	*Adj R^2^*	*AIC*	*Δ*°*AIC*	*logLik*	*RMSE*	*R^2^*	*Adj R^2^*
Model 1	968.7	0	−480.35	8.72	0.01	0.01	989.2	0	−490.59	9.41	0.01	0.01
Model 2	961.9	6.8	−475.95	8.44	0.08	0.05	982.6	6.6	−486.31	9.12	0.07	0.04
Model 3	961.2	7.5	−474.58	8.35	0.09	0.08	983.4	5.8	−485.69	9.08	0.08	0.05
Model 4	952.0	16.7	−465.99	7.83	0.20	0.15	**957.1**	**32.1**	−468.55	7.98	0.28	0.24
Model 5	**951.7**	**17**	**−460.84**	**7.54**	**0.26**	**0.18**	960.4	28.8	**−465.22**	**7.79**	**0.32**	**0.25**

GAD index, Generalized Anxiety Disorder index; AIC, Akaike Information Criterion; Δ°AIC, difference in AIC with respect to the first model; RMSE, Root Mean Square Error. The lower the AIC and the RMSE, the higher the logLik and the R^2^ and Adj R^2^, the better the model. Bold values represent the best indexes.

**Figure 1:**
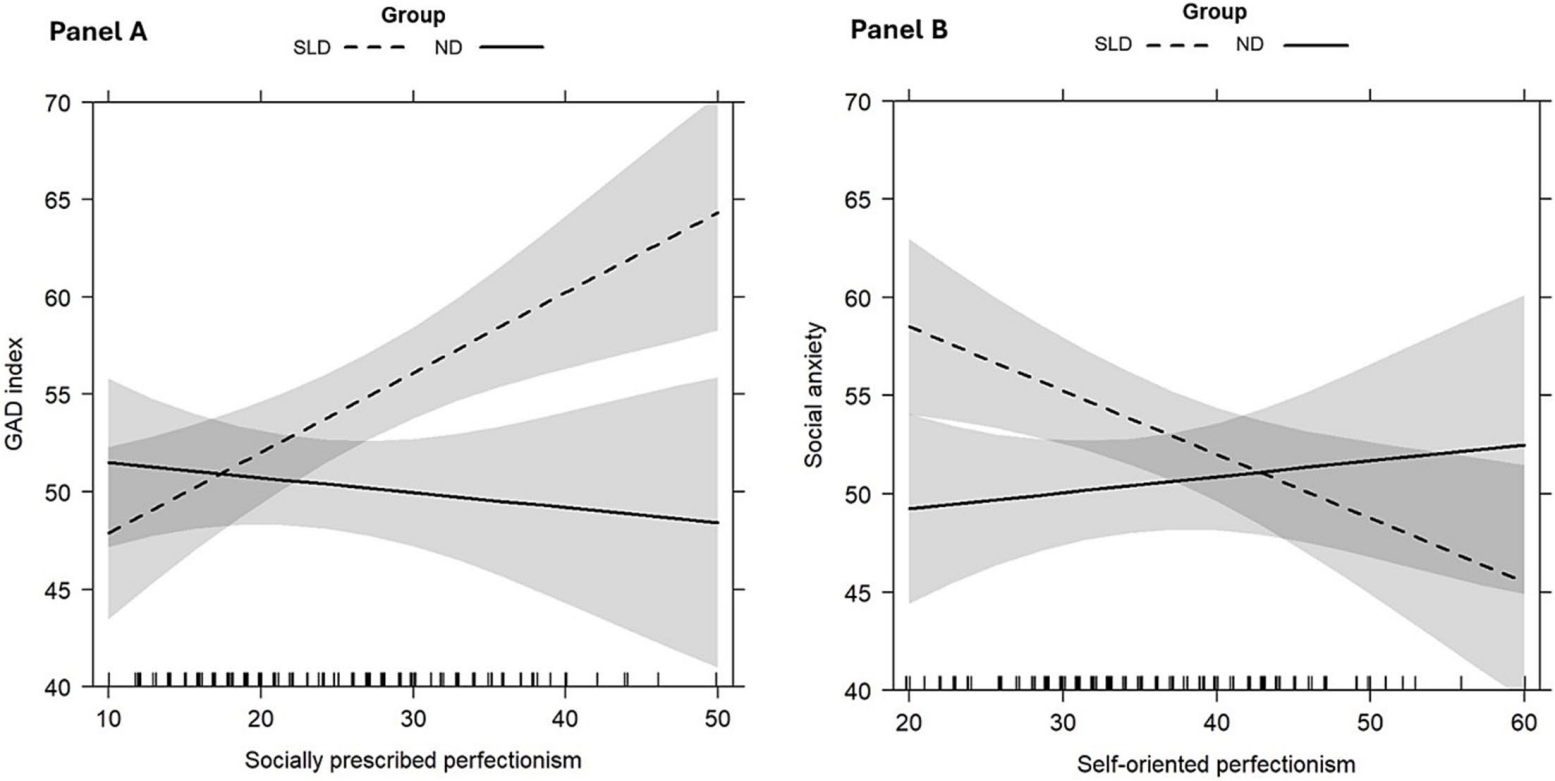
Significant interaction effects of Models 5: socially prescribed perfectionism*group on GAD index **(A)**, and self-oriented perfectionism*group on social anxiety **(B)**. Error bands represent 95% confidence intervals.

Our model fitting procedure revealed that the best-fitting model with GAD index as dependent variable was Model 5 (*AIC* = 951.7, *Δ°AIC* = 17, *logLik* = −460.84, *RMSE* = 7.54, *R^2^ = 0*.26*, Adj R^2^ = 0*.18). The *Δ°AIC* reflects the difference in AIC with respect to the first step with only covariates. Taken together, our variables in the final model accounted for 26% of the variance calculated using the *R^2^* (*Adj R^2^* = 0.18), adding around 25% of variance to the first model which included only age and sex. An interaction effect was found to be statistically significant between socially prescribed perfectionism and group, *β =* −0.49*, t =* −2.67*, p = 0*.008. As shown in Figure 1A, higher levels of socially prescribed perfectionism were associated with higher levels of GAD index in the SLD group.

As concerns social anxiety, the best-fitting model was Model 5 as well. Despite the *AIC* being slightly lower in Model 4, the other fit indices indicate that Model 5 is superior (Model 4: *AIC* = 957.1, *Δ°AIC* = 32.1, *logLik* = −468.55, *RMSE* = 7.98, *R^2^ = 0*.28*, Adj R^2^ = 0*.24; Model 5: *AIC* = 960.4, *Δ°AIC* = 28.8, *logLik* = −465.22, *RMSE* = 7.79, *R^2^ = 0*.32*, Adj R^2^ = 0*.25). Taken together, our variables in Model 5 accounted for 32% of the variance calculated using the *R^2^* (*Adj R^2^* = 0.25), adding around 32% of variance to the first model which included only age and sex. Three main effects were found to be statistically significant: higher levels of self-oriented perfectionism, *β =* −0.32*, t =* −2.74*, p = 0*.007, lower levels of socially prescribed perfectionism, *β = 0*.33*, t =* 2.70*, p = 0*.008, and shame, *β = 0*.46*, t =* 2.94*, p = 0*.004, were consistent with lower reported social anxiety in the whole sample. Moreover, as presented in Figure 1B, an interaction effect between self-oriented perfectionism and group was found to be statistically significant, *β = 0*.41*, t =* 2.17*, p = 0*.03: higher levels of self-oriented perfectionism were related to lower levels of social anxiety in the SLD group.

## Discussion

The current study aimed to investigate the possible underlying mechanisms associated with the occurrence of generalized and social anxiety in young people with SLD compared to non-diagnosed peers, focusing on cognitive factors (i.e., inhibitory control), personality dispositions (i.e., self-oriented and socially prescribed perfectionism), and self-conscious emotions (i.e., shame and guilt).

Consistent with previous research findings and with our initial hypothesis, participants with SLD reported higher levels of anxiety than non-diagnosed peers (Nelson and Harwood, 2011). Specifically, participants with SLD seem to experience higher symptom levels for both generalized and social anxiety (Carroll et al., 2005; Carroll and Iles, 2006; Goldston et al., 2007; Mammarella et al., 2016; Thaler et al., 2010), highlighting a crucial phenomenon to consider both in the assessment and the intervention of this clinical condition. Anxiety symptoms reported by participants with SLD include severe fear of negative evaluation, avoidance of social interactions, but also pervasive worries that concretized in safety behaviors (e.g., keeping the light on at night) and physical signs (e.g., tension and gastrointestinal discomfort). These indicators may stem from repeated experiences of academic difficulties, criticism, or misunderstanding by others, which can erode self-esteem and foster a persistent worry of failure (Alexander-Passe, 2006; Filippello et al., 2020; Mammarella et al., 2016; Novita, 2016; Zuppardo et al., 2023). Puberty and adolescence, on the other hand, represent extremely delicate periods regarding mood regulation and social adaptation, and it could be even more so for people with SLD (Giovagnoli et al., 2020), who could also have troubles with social interactions and friendship stability (Wiener and Schneider, 2002; Wilmot et al., 2024).

Regarding our second aim, focused on vulnerability factors for anxiety, our hypotheses have been partially confirmed. Participants with SLD made more errors in the inhibitory control task, demonstrating lower ability to hinder an impulsive response and avoid distractions (Agostini et al., 2022; Capodieci et al., 2023; Crisci et al., 2021; Mirabella, 2021). This weakness can be particularly disabling in school settings, where following certain rules is required in completing educational tasks. In this regard, social expectations (for example, from teachers and parents) may represent an additional vulnerability for participants with SLD. In fact, our results show a greater tendency toward socially prescribed perfectionism (Stoeber and Rountree, 2021), as well as stronger feelings of shame (Alexander-Passe, 2006; Gibby-Leversuch et al., 2021) in individuals with SLD compared to their peers without a diagnosis. Repeated academic struggles, perceived failure to meet external standards, and a heightened sensitivity to social comparison, can lead students with SLD to internalize others’ expectations and feel inadequate in different settings. However, contrary to our expectations, we did not find any differences between students with and without SLD in guilt-proneness, suggesting that shame could represent a more prominent retrospective outcome emotion possibly linked to failure at school (Pekrun, 2006; Sullins et al., 2024).

Our third aim was to examine whether and how these vulnerability mechanisms might be associated with the levels of generalized and social anxiety in the considered groups, controlling for age and sex. While the study’s cross-sectional design inherently limits causal inferences, it still sheds light on important associations between anxiety and related factors, providing valuable insights into the emotional and cognitive challenges faced by children with SLD. Regarding cognitive factors, our results did not show a relationship between inhibitory control and anxiety symptoms, confirming previous findings (for a review, see Oosterlaan et al., 1998). However, it may be essential to consider the role of different executive functions in understanding how certain cognitive vulnerabilities contribute to the onset of anxiety (Zainal and Newman, 2018). In fact, people with SLD have been reported to have greater problems with attentional skills (Franceschini et al., 2022; Sterr, 2004), and working memory (Peng and Fuchs, 2016; Toffalini et al., 2017). Thus, it may be critical to consider executive functions other than inhibition when the investigating the underlying aspects of anxiety in SLD.

On the contrary, personality dispositions and self-conscious emotions appear to play an important role in both generalized and social anxiety symptoms, but with different patterns across participants with and without SLD. Specifically, socially prescribed perfectionism significantly predicted the levels of generalized anxiety in individuals with SLD, representing a hallmark for this clinical group. Indeed, worry driven by the belief that others expect perfection can generalize and lead to extensive negative consequences in young people with SLD (Klibert et al., 2015). In this way, social standards may not only pose a risk factor for the development of social anxiety symptoms, but also contribute to persistent, excessive, and unrealistic worry about everyday situations in participants with SLD. From this standpoint, youth with SLD with high levels of socially prescribed perfectionism may create more stress for themselves by perceiving greater levels of harm in minor life events (Klibert et al., 2015; Klibert et al., 2005).

Not surprisingly, socially prescribed perfectionism and shame were significant predictors of social anxiety in the whole sample. A recent meta-analysis (Ferber et al., 2024) showed large to very-large-sized associations between social anxiety and dimensions of perfectionism, including socially prescribed perfectionism. As a consequence of perceived inadequacy, people may closely monitor their social performance and be highly critical of their apparent flaws, developing feelings of shame (Swee et al., 2021). In fact, a previous study (Swee et al., 2021) have shown a bidirectional relationship between shame and fear of negative evaluation, with shame contributing to post-event processing and avoidance behaviors that can sustain social anxiety symptoms.

Instead, self-oriented perfectionism seemed to be associated with lower social anxiety symptoms in participants with SLD. This result confirms and expands upon previous findings regarding the potential adaptive role of self-oriented perfectionism, in contrast to the socially prescribed one (Osenk et al., 2020; Stoeber et al., 2009; Stoeber and Rountree, 2021; Wheeler et al., 2011). Self-oriented perfectionism has been shown to be associated with intrinsic motivation for studying with lower interference and higher confidence in tests (Stoeber et al., 2009). In this sense, setting high standards for oneself can have a crucial effect on academic outcomes (Osenk et al., 2020). The underlying mechanism might be that students with SLD who exhibit higher self-oriented perfectionism tend to have a more optimistic view of their abilities, as they set higher personal goals that motivates them. This focus on their own skills, rather than on others’ judgments, could act as a protective barrier against developing a fear of negative evaluation and social concerns. However, the role of other factors might be considered in the relationship between self-oriented perfectionism and social anxiety, such as self-efficacy, coping strategies, and the presence of a supportive learning environment. For students with SLD, this combination might foster a more optimistic self-perception, as they focus on personal growth rather than external validation.

The current study presents some limitations. One limitation of our study concerns the selection of the SLD sample, and the exclusion criteria applied. Excluding participants with comorbid conditions or psychotropic medication use improves internal validity but may limit the generalizability of our findings to real-world SLD populations, where such comorbidities are common. While this approach allows for a more precise analysis of specific relationships, it may not fully capture the complexity of SLD in applied settings. Future research should consider the impact of comorbidities to enhance the ecological validity of findings. In addition, in the selection of SLD participants, due to the need to balance the availability of clinicians and families, along with the strict inclusion and exclusion criteria, assessing a larger sample of SLD participants was not feasible. While this limitation did not appear to affect the study’s primary outcomes, future research should prioritize recruiting larger and more diverse samples to improve statistical power and enable broader generalizations. Second, we considered only inhibition as a cognitive variable, excluding other executive functions (e.g., working memory) that might be more predictive of anxiety in individuals with SLD (Wang et al., 2024). Moreover, various studies have highlighted the role of behavioral inhibition, rather than cognitive inhibition in the development of anxiety (Thorell et al., 2004; White et al., 2011). Future research could explore the combined effect of temperamental and cognitive factors in the etiology of anxiety in SLD. Third, we examined perfectionism based on Flett and co-authors’ (2016) categorization of self-oriented and socially prescribed dimensions. However, it would be interesting to investigate the contribution of perfectionism to anxiety in SLD by also considering Hamachek (1978) classification, by separating healthy perfectionists (high perfectionistic strivings, low perfectionistic concerns), unhealthy perfectionists (high perfectionistic strivings, high perfectionistic concerns), and non-perfectionists (low perfectionistic strivings). However, this was prevented by our sample size; thus, future research should collect a considerable number of participants with SLD to be able to run this type of investigation. Moreover, our sample includes only Italian-speaking youth, which may restrict the generalizability of the findings to other cultural and linguistic contexts. Future research should examine whether similar results emerge in diverse linguistic and cultural settings to determine the potential cross-cultural differences in the studied constructs. Also, the methodology relies exclusively on subjective self-reports from children. While self-reports provide valuable insights into children’s experiences, the integration of multiple measures, such as behavioral measures, parents’ and teachers’ reports, might strengthen future research on this topic.

More importantly, anxiety is a multifaceted construct shaped by both individual and environmental factors, and it is crucial to examine how socio-cultural contexts interact with the severity of learning difficulties to influence emotional well-being. Research suggests that individuals with different types of learning difficulties (e.g., reading, math) seem to experience different levels and kinds of anxiety (Aro et al., 2022; Francis et al., 2019, 2022; Polak and Grossman, 2024). Moreover, heightened emotional distress may stem from the broader impact of multiple learning difficulties on self-esteem, leading to diminished adaptive self-regulation skills, increased frustration and anxiety in learning situations (Aro et al., 2022). Beyond cognitive profiles, environmental factors also play a key role in shaping (Brunelle et al., 2020; Macdonald and Deacon, 2019; Nevill and Forsey, 2023), shame and guilt (Luo et al., 2025; Muris and Meesters, 2014; Stearns and Stearns, 2017) in children with SLD. Socio-economic and cultural contexts affect how children perceive and cope with their difficulties, with access to educational resources, specialized interventions, and supportive school environments serving as protective factors (Grigorenko et al., 2020). Conversely, children from lower socio-economic backgrounds may face heightened anxiety due to limited academic support and increased stressors (Brunelle et al., 2020). Given these complexities, future research should further investigate how the interaction between severity levels and socio-economic status influences cognitive and emotional outcomes in SLD populations, offering a more comprehensive understanding of anxiety in these children.

Despite the limitations, our findings could have both educational and clinical implications. Educators should keep in mind how anxiety symptoms can significantly impact both the learning process of students with academic difficulties and their self-concept and perceived self-efficacy. The academic development of young people with SLD may be supported by preventing feelings of inadequacy in the classroom, such as shame, which arise from negative experiences, criticism, and the sense of being “different.” Moreover, both teachers and parents should be aware that social expectations may trigger maladaptive coping strategies in youth with SLD. From a clinical perspective, investigating the underlying mechanisms behind the onset of generalized and social anxiety symptoms in individuals with SLD is crucial for earlier identification and more targeted interventions. Our study further highlights the importance of reassessing current practices aimed at improving the well-being of students with SLD. Historically, interventions have primarily concentrated on strengthening academic abilities (Fletcher et al., 2018). However, the experiences shared by our participants indicate that coping strategies, personality dispositions and self-conscious emotions should be considered as well in the assessment and intervention of students with SLD, rather than academic skills alone. Practical evidence-based techniques, such as cognitive-behavioral practices, can help students manage anxiety by challenging negative thought patterns, and empowering a more positive mindset (Seligman and Ollendick, 2011). Mindfulness and stress-reduction practices (e.g., breathing exercises, progressive muscle relaxation, or short mindfulness breaks) may facilitate students develop greater awareness of their emotions improving emotional regulation (Fulambarkar et al., 2023). Explicit self-regulation training, such as breaking tasks into smaller steps and using planners, can enhance executive functioning, which is essential for academic success (Putwain, 2019). When students have clear expectations and receive clear instructions with manageable steps, they feel more in control and engaged in learning. Finally, school-based social–emotional learning programs strengthen coping skills, self-control and resilience (Elbertson et al., 2009). By integrating these approaches, students with SLD can develop the skills needed to navigate academic and emotional challenges more effectively.

To conclude, young people with SLD seem to experience greater symptoms of both generalized and social anxiety as compared to non-diagnosed peers. While the two groups differ in inhibitory control skills, socially prescribed perfectionism and shame, our study highlights the possible different roles of personality dispositions and self-conscious emotions in determining anxiety levels. On the one hand, socially prescribed perfectionism represents a risk factor for generalized anxiety in youth with SLD. On the other hand, socially prescribed perfectionism and shame significantly predict social anxiety in both groups, while self-oriented perfectionism seems to be associated with social anxiety symptoms in participants with SLD.

## Data Availability

The raw data supporting the conclusions of this article will be made available by the authors, without undue reservation.
