# Supplementary material for: Anxiety in youth with and without specific learning disorders: exploring the relationships with inhibitory control, perfectionism, and self-conscious emotions
Source: Front Behav Neurosci. 2025 Mar 10;19:1536192. doi: 10.3389/fnbeh.2025.1536192 (PMC11931035; doi:10.3389/fnbeh.2025.1536192)
Supplement: Supplementary file 1 [file Table_1.docx]

**Correlations**

Considering the SLD group, the GAD index shows a significant positive correlation with social anxiety, *r*=.69, *p*<.001, socially prescribed perfectionism, *r*=.34, *p*<.01, and shame, *r*=.29, *p*<.05, whereas social anxiety positively correlates with socially prescribed perfectionism, *r*=.28, *p*<.05, shame, *r*=.43, *p*<.001, and guilt, *r*=.30, *p*<.05. Self-oriented perfectionism positively correlates with guilt in the SLD group, *r*=34, *p*<.01.

As regard the ND group, the GAD index shows a significant positive correlation with social anxiety, *r*=.67, *p*<.001, and shame, *r*=.32, *p*<.01, while social anxiety positively correlates with socially prescribed perfectionism, *r*=.27, *p*<.05, and shame, *r*=.36, *p*<.01. In this group, self-oriented perfectionism is positively associated with socially prescribed perfectionism, *r*=.30, *p*<.05, and guilt, *r*=.33, *p*<.01.

Shame and guilt positively correlate in both groups, SLD: *r*=.69, *p*<.001, ND: *r*=.39, *p*<.01. Table S1 shows the Spearman’s correlations between all variables divided by group (SLD, ND).

**Table S1.** Spearman’s correlations on measured variables divided by group (SLD in the lower diagonal, and ND in the upper diagonal).

| **Variables** | **1.** | **2.** | **3.** | **4.** | **5.** | **6.** | **7.** |
| --- | --- | --- | --- | --- | --- | --- | --- |
| 1. **GAD index** | - | .67*** | -.16 | .19 | .05 | .32*** | .21 |
| 1. **Social anxiety** | .69*** | - | -.21 | .21 | .27* | .36** | .07 |
| 1. **Inhibitory control** | -.13 | .01 | - | -.06 | -.16 | -.21 | .33** |
| 1. **Self-oriented perfectionism** | -.04 | -.18 | -.12 | - | .30* | .21 | .33** |
| 1. **Socially prescribed perfectionism** | .34** | .28* | .03 | .04 | - | .23 | -.11 |
| 1. **Shame** | .29* | .43*** | .01 | .18 | .02 | - | .39** |
| 1. **Guilt** | .21 | .30* | .04 | .34** | -.01 | .69*** | - |

*Notes:* GAD index, Generalized Anxiety Disorder index. *p*<.001***, *p*<.01**, *p*<.05*.
